# GDF-15 for Prognostication of Cardiovascular and Cancer Morbidity and Mortality in Men

**DOI:** 10.1371/journal.pone.0078797

**Published:** 2013-12-02

**Authors:** Lars Wallentin, Björn Zethelius, Lars Berglund, Kai M. Eggers, Lars Lind, Bertil Lindahl, Kai C. Wollert, Agneta Siegbahn

**Affiliations:** 1 Uppsala Clinical Research Center (UCR) and Department of Medical Sciences, Uppsala University, Uppsala, Sweden; 2 Department of Public Health/Geriatrics, Uppsala University and Medical Products Agency/Epidemiology, Uppsala, Sweden; 3 Department of Medical Sciences, Uppsala University, Uppsala, Sweden; 4 Division of Molecular and Translational Cardiology, Department of Cardiology and Angiology, Hannover Medical School, Hannover, Germany; University of Oxford, United Kingdom

## Abstract

The objective was to evaluate the hypothesis that growth-differentiation factor 15 (GDF-15) is an independent marker of the long-term risk for both cardiovascular disease and cancer morbidity beyond clinical and biochemical risk factors. Plasma obtained at age 71 was available from 940 subjects in the Uppsala Longitudinal Study of Adult Men (ULSAM) cohort. Complete mortality and morbidity data were obtained from public registries. At baseline there were independent associations between GDF-15 and current smoking, diabetes mellitus, biomarkers of cardiac (high-sensitivity troponin-T, NT-proBNP) and renal dysfunction (cystatin-C) and inflammatory activity (C-reactive protein), and previous cardiovascular disease (CVD). During 10 years follow-up there occurred 265 and 131 deaths, 115 and 46 cardiovascular deaths, and 185 and 86 events with coronary heart disease mortality or morbidity in the respective total cohort (n=940) and non-CVD (n=561) cohort. After adjustment for conventional cardiovascular risk factors, one SD increase in log GDF-15 were, in the respective total and non-CVD populations, associated with 48% (95%CI 26 to 73%, p<0.001) and 67% (95%CI 28 to 217%, p<0.001) incremental risk of cardiovascular mortality, 48% (95%CI 33 to 67%, p<0.001) and 61% (95%CI 38 to 89%, p<0.001) of total mortality and 36% (95%CI 19 to 56%, p<0.001) and 44% (95%CI 17 to 76%, p<0.001) of coronary heart disease morbidity and mortality. The corresponding incremental increase for cancer mortality in the respective total and non-cancer disease (n=882) population was 46% (95%CI 21 to 77%, p<0.001) and 38% (95%CI 12 to 70%, p<0.001) and for cancer morbidity and mortality in patients without previous cancer disease 30% (95%CI 12 to 51%, p<0.001). In conclusion, in elderly men, GDF-15 improves prognostication of both cardiovascular, cancer mortality and morbidity beyond established risk factors and biomarkers of cardiac, renal dysfunction and inflammation.

## Introduction

 Established risk factors predict about two thirds of future CVD events in community-dwelling individuals [1]. Several of these risk factors for CVD, including age, smoking, obesity, and diabetes, are also related to cancer morbidity and mortality [2]. We and others recently found that a combination of troponin I, N-terminal pro-B-type natriuretic peptide (NT-proBNP), cystatin-C, and C-reactive protein (CRP) provided incremental prognostic information concerning cardiovascular mortality [3,4], which might be even further improved by more sensitive troponin assays [5,6]. 

 Growth-differentiation factor-15 (GDF-15) is a distant member of the transforming growth factor-beta cytokine superfamily. The expression of GDF-15 increases in response to oxidative stress and inflammation in cardiovascular cells and tumour cells [7]. In community-dwelling individuals and in patients with established CVD, increased levels of GDF-15 are related to cardiovascular risk factors, inflammatory activity, and estimates of impaired cardiovascular and renal function [7-14]. GDF-15 has emerged as a strong and independent predictor of all-cause and cardiovascular mortality in patients with heart failure and different manifestations of ischemic heart disease [15-18]. The GDF-15 level is also elevated in several cancers including prostate cancer, ovarian cancer, pancreatic cancer, colorectal cancer, and multiple myeloma [7,19-25]. In some cancer types, elevated levels of GDF‑15 have been associated with an adverse prognosis [22,23]. Recently several studies have found GDF-15 prognostic for long-term cardiovascular and non-cardiovascular mortality in healthy subjects without previous CVD [26-30], and in one of these studies [26], a high GDF-15 level was related to both cardiovascular and cancer mortality. 

 The present study evaluated the hypothesis that GDF-15 is an independent marker of the long-term risk for both cardiovascular disease and cancer morbidity beyond clinical and biochemical risk factors in elderly men, with and without previous manifestations of these diseases.

## Material and Methods

### Study population

 The study population came from the Uppsala Longitudinal Study of Adult Men (ULSAM), which was initiated in 1970, when all men born between 1920 and 1924 living in Uppsala, Sweden, were invited to a health survey (www.pubcare.uu.se/ULSAM). The present analyses were based on the baseline examination when participants were approximately 71 years of age. This population has thereafter been followed for a median of 9.8 years (range 0.1 -12.4 years). Of the 1221 participants, 940 had baseline plasma samples available for simultaneous measurements of biochemical markers. All participants gave written informed consent, and the ethics committee at the Faculty of Medicine of Uppsala University approved the study.

### Baseline measurements

 Information on clinical history and smoking status (current smoker *vs.* non-smoker) was obtained from a questionnaire. Participants’ smoking habits, body weight, body mass index (BMI), and waist circumference was obtained at the baseline visit. Obesity was defined as BMI ≥30 kg/m^2^. Systolic and diastolic blood pressures were measured, and a 12-lead electrocardiogram was obtained with the participant in a supine position. Plasma glucose (fasting and 120 minutes after an oral glucose load) and fasting serum total, LDL and HDL cholesterol levels were measured by routine laboratory analyses. Type 2 diabetes mellitus was defined by fasting plasma glucose >7 mmol/L (corresponding to >126 mg/dL), or the use of oral hypoglycemic agents or insulin. 

### Biochemical methods

 For biomarker measurements, venous blood samples were drawn at baseline and stored at –70°C for a median of 16.5 years (range 14.8-18.5) prior to analysis. CRP was assayed with the use of latex-enhanced reagents (Siemens), on a BN ProSpec analyzer (Siemens). The high sensitivity Troponin T (Lot number 153 401), NT-proBNP, GDF-15 and Cystatin C analyses were determined with sandwich immunoassays on Cobas Analytics immunoanalyzers (Roche Diagnostics). GDF-15 was measured with a pre-commercial assay (Roche Diagnostics) using a monoclonal mouse antibody for capture and a monoclonal mouse antibody fragment (F(ab´)2) for detection in a sandwich assay format. Detection was based on an electrochemiluminescence immunoassay (ECLIA), using a ruthenium(II) complex label. The pre-commercial assay correlates closely with a previously established IRMA method [10] (r=0.98, regression Passing/Bablok: slope 1.049, intercept -136 ng/L). The assay has an inter-assay coefficient of variation of 2.3% at 1100 ng/L and 1.8% at 17200 ng/mL, an intra-assay coefficient of variation of 0.8% at 1100 ng/L and 0.9% at 18600 ng/mL, and a lower detection limit of 10 ng/L. 

### Cardiovascular and cancer mortality and morbidity

 Cardiovascular disease (CVD) was defined by the following criteria: prior myocardial infarction or angina pectoris, Q or QS waves or left bundle-branch block (Minnesota codes 1.1 to 1.3 and 7.1, respectively) on the baseline electrocardiogram; current treatment with nitroglycerin or cardiac glycosides, or a history of any CVD, as noted in the national registries. Complete information on mortality and morbidity from all patients were collected from the Swedish Cause of Death and Hospital Admission registries. The events were classified as CVD mortality (ICD-10 codes I00-I99), coronary heart disease (CHD) mortality or morbidity (ICD-10 codes I20-I25), stroke mortality or morbidity (ICD-10 codes I60-I69). Cancer disease was defined as any diagnosis of malignancy in the same registries (ICD-10 codes C00-D48). Concerning cancer, only the first non-fatal event was recorded; accordingly, cancer morbidity after the baseline visit only could be evaluated in participants without any previous cancer diagnosis at baseline. All clinical endpoints were classified as either previous or incident disease at the baseline visit or as occurring during follow-up.

### Statistics

 The study aimed at investigating the relations between GDF-15 and previous (cross-sectional) and subsequent (longitudinal) manifestations of cardiovascular and cancer disease, and mortality, both in the total population (n=940), and in subgroups without CVD (n=561) and without cancer disease (n=882) at baseline. The aims of the cross-sectional analyses were to investigate the relations between GDF-15 and established risk factors for the previous occurrence of CVD and cancer until the age 71. The aims of the longitudinal analyses were to investigate if GDF-15 provided incremental value beyond established risk factors and/or other biomarkers in predicting CVD and cancer during long-term follow-up. 

 Continuous variables were described with means and standard deviations. For continuous variables the Shapiro-Wilk’s test statistic W was calculated where the region W ≥0.95 with a respective original or logarithmic scale led to the use of a parametric method while otherwise a non-parametric method was used. All statistical tests and confidence intervals were two-sided (where applicable), and the region p<0.05 was used to declare a statistically significant result without adjustments for multiplicity. The univariate associations between the continuous risk factors (including other biomarkers) and GDF-15 were assessed with the parametric Pearson correlation, if both variables were normally distributed possibly after logarithmic transformation, and otherwise with the non-parametric Spearman rank correlation coefficients. The univariate associations between co-morbidities and GDF-15, and associations adjusting for cardiovascular risk factors, were assessed with logistic regression models. Results from the logistic regression models were presented as odds ratios, with 95% confidence intervals, of a one standard deviation (SD) increase of log GDF-15, and p values.

 The linearity of the relation between continuous predictors and the longitudinal outcome events were examined visually in GAM plots and, in case of a non-normal distribution or non-linear relation, a logarithmic transformation was used. Proportional-hazards assumptions of Cox regression models were confirmed with Schoenfeld’s test. The longitudinal analyses were investigated with Cox proportional-hazards regression models and presented, for each continuous predictor, as hazard ratios, with 95% confidence intervals, of a one standard deviation increase of the predictor, and p values. Discriminative abilities of the models were estimated as C statistics for Cox regression models according to Pencina [31]. The increased discriminative ability of one regression model *vs.* another model was estimated based on the difference between two models in the individual estimated probability from logistic regression models using as measure the integrated discrimination improvement (IDI) and the continuous net reclassification index (NRI) according to Pencina [32,33]. The used Cox regression models included as predictors each of the following variables alone or in several different combinations: established risk factors (age, current smoking, BMI, total cholesterol, HDL cholesterol, lipid-lowering treatment, systolic blood pressure, antihypertensive treatment, type 2 diabetes, cancer before or at age 71), troponin T, GDF-15, NT-proBNP, CRP, and cystatin C. Kaplan-Meier curves (showing one minus event probability) were also presented stratified on tertiles of the biomarker.

## Results

### Clinical characteristics and biomarkers at baseline

 Clinical characteristics, concomitant diseases, the average levels of conventional risk factors, GDF-15, and other tested biomarkers in the total material of 940 men at the baseline investigation, are presented in Table 1. As expected there were slightly lower average levels of all risk factors and biomarkers in the 561 men without previous manifestation of CVD. The baseline characteristics in the 882 men without previous cancer were similar to the total population.

**Table 1:** Baseline characteristics; measurements and concurrent diseases in the whole sample, subjects without cardiovascular disease and subjects without cancer disease until age 71 (values are mean (SD) unless otherwise stated).

**Variable**	**Whole sample (n=940)**	**Men without CVD (n=561)**	**Men without cancer (n=882)**
Age	71.0 (0.7)	71.0 (0.6)	71.0 (0.7)
Current smoking n (%)	194 (21)	117 (21)	186 (21)
BMI	26.2 (3.4)	25.9 (3.1)	26.2 (3.4)
Obesity (BMI >= 30 kg/m^2^)	111 (12)	50 (9)	102 (12)
Waist girth (cm)	94.4 (9.5)	93.7 (9.2)	94.4 (9.5)
Total cholesterol (mmol/l)	5.78 (0.99)	5.76 (0.98)	5.78 (0.99)
LDL cholesterol (mmol/l)	3.86 (0.88)	3.84 (0.87)	3.87 (0.88)
HDL cholesterol (mmol/l)	1.29 (0.35)	1.32 (0.36)	1.29 (0.35)
S-Triglycerides (mmol/l)	1.41 (0.74)	1.34 (0.73)	1.40 (0.75)
Lipid-lowering treatment n (%)	87 (9)	38 (7)	85 (10)
SBP (mmHg)	146.6 (19.1)	147.0 (18.8)	146.8 (19.1)
DBP (mmHg)	83.5 (9.6)	83.9 (9.5)	83.7 (9.6)
Antihypertensive treatment n (%)	323 (34)	130 (23)	297 (34)
Hypertension n (%)	696 (74)	397 (71)	652 (74)
Fasting glucose	5.76 (1.45)	5.67 (1.32)	5.76 (1.45)
120 min glucose	8.37 (4.15)	8.07 (3.90)	8.35 (4.13)
Type 2 diabetes n (%)	101 (11)	47 (8)	94 (11)
Troponin T (ng/l)	10.3 (7.6)	9.1 (6.1)	10.2 (7.7)
GDF-15 (ng/l)	1677 (809)	1582 (681)	1677 (815)
proBNP (ng/l)	212.7 (405.7)	129.1 (207.7)	210.5 (404.6)
CRP (mg/l)	3.4 (4.8)	3.2 (4.4)	3.3 (4.6)
Cystatin C (mg/l)	1.07 (0.22)	1.04 (0.19)	1.06 (0.22)
Cardiovascular disease (CVD) n (%)	379 (40)	0 (0)	349 (40)
Coronary heart disease (CHD) n (%)	115 (12)	1 (0)	109 (12)
Stroke n (%)	30 (3)	0 (0)	29 (3)
CVD, CHD or stroke n (%)	380 (40)	1 (0)	350 (40)
Cancer n (%)	58 (6)	28 (5)	0 (0)
Prostate cancer n (%)	24 (3)	13 (2)	0 (0)

### GDF-15 and its relations to biomarkers and co-morbidities at baseline

 In the total population, the median GDF-15 level was 1494 ng/L (25^th^ and 75^th^ percentiles, 1216-1882 ng/L). The associations between baseline characteristics and tertiles of GDF-15 levels are shown in Table 2, and significances of the adjusted correlations in Table 3 and table 4. There were significant independent associations between GDF-15 levels and current smoking, diabetes mellitus, and biomarkers indicating renal dysfunction (cystatin C), cardiac dysfunction (NT-proBNP, troponin T) and also inflammatory activity (CRP). There were also significant independent relations between GDF-15 and previous cardiovascular or coronary heart disease and stroke morbidity, but not with previous cancer.

**Table 2:** Baseline characteristics; measurements and concurrent diseases by tertiles of GDF-15 in the whole sample until age 71 (values are mean (SD) unless otherwise stated).

Variable	GDF-15 tertile 1: <1307 ng/L (n = 314)	GDF-15 tertile 2: 1307-1720 ng/L (n = 313)	GDF-15 tertile 3: >1720 ng/L (n = 313)
Age	70.9 (0.6)	71.0 (0.7)	71.0 (0.6)
Current smoking n (%)	31 (10)	68 (22)	95 (30)
BMI	26.1 (3.1)	26.2 (3.6)	26.2 (3.5)
Obesity (BMI >= 30 kg/m^2^)	28 (9)	40 (13)	43 (14)
Waist girth (cm)	94.2 (8.9)	94.3 (9.7)	94.8 (10.1)
Total cholesterol (mmol/l)	5.86 (0.95)	5.75 (1.02)	5.71 (0.99)
LDL cholesterol (mmol/l)	3.94 (0.84)	3.83 (0.93)	3.81 (0.88)
HDL cholesterol (mmol/l)	1.30 (0.33)	1.32 (0.38)	1.24 (0.34)
S-Triglycerides (mmol/l)	1.40 (0.76)	1.35 (0.66)	1.48 (0.80)
Lipid-lowering treatment n (%)	27 (9)	30 (10)	30 (10)
SBP (mmHg)	144.9 (17.8)	147.2 (19.4)	147.6 (20.0)
DBP (mmHg)	82.7 (9.2)	83.8 (10.0)	84.1 (9.6)
Antihypertensive treatment n (%)	76 (24)	115 (37)	132 (42)
Hypertension n (%)	213 (68)	241 (77)	242 (77)
Fasting glucose	5.69 (1.31)	5.61 (1.10)	5.99 (1.82)
120 min glucose	8.00 (3.80)	7.98 (3.28)	9.15 (5.08)
Type 2 diabetes n (%)	28 (9)	16 (5)	57 (18)
Troponin T (ng/l)	8.7 (5.1)	9.1 (5.4)	12.9 (10.4)
GDF-15 (ng/l)	1102 (140)	1500 (116)	2431 (1002)
proBNP (ng/l)	128.5 (181.8)	189.9 (285.3)	319.9 (600.9)
CRP (mg/l)	2.6 (2.8)	3.4 (5.7)	4.1 (5.3)
Cystatin C (mg/l)	0.97 (0.15)	1.06 (0.17)	1.16 (0.27)
Cardiovascular disease (CVD) n (%)	104 (33)	127 (41)	148 (47)
Coronary heart disease (CHD) n (%)	29 (9)	37 (12)	49 (16)
Stroke n (%)	3 (1)	10 (3)	17 (5)
CVD, CHD or stroke n (%)	104 (33)	127 (41)	149 (48)
Cancer n (%)	20 (6)	17 (5)	21 (7)
Prostate cancer n (%)	6 (2)	5 (2)	13 (4)

**Table 3:** Cross-sectional associations between GDF-15 levels and previous or current occurrence of obesity, smoking, type 2 diabetes, cardiovascular disease (CVD), coronary heart disease (CHD), stroke, cancer or prostate cancer in the whole sample (n = 940), with unadjusted and adjusted odds ratios (OR) with 95 % CI and p values for 1 SD increase of log GDF-15 for these dichotomous risk factors and co-morbidities.

Risk factors or co-morbidities	Unadjusted OR	95 % CI	p value	Adjusted^1^ OR	95 % CI	p value
Obesity	1.22	1.02,1.47	0.033	1.08	0.88,1.31	0.48
Current smoking	1.55	1.33,1.80	<0.001	1.60	1.37,1.88	<0.001
Type 2 diabetes	1.57	1.30,1.88	<0.001	1.53	1.26,1.86	<0.001
CVD	1.38	1.20,1.58	<0.001	1.33	1.15,1.53	<0.001
CHD	1.40	1.17,1.58	<0.001	1.34	1.11,1.61	0.002
Stroke	1.62	1.22,2.16	<0.001	1.76	1.30,2.38	<0.001
CVD,CHD, stroke	1.38	1.21,1.58	<0.001	1.34	1.16,1.54	<0.001
Cancer	1.00	0.77,1.31	0.99	1.00	0.76,1.32	1.00
Prostate cancer	1.32	0.93,1.88	0.12	1.30	0.91,1.87	0.15

^1^ Adjusted for hypertension, type 2 diabetes, and (where applicable) current smoking, LDL and HDL cholesterol.

**Table 4:** Cross-sectional associations between GDF-15 levels and measurements and biomarker levels determined at baseline in the whole sample (n = 940), with unadjusted and adjusted correlation coefficients and p values for these continuous variables.

Variable Measurements at baseline	Unadjusted		Adjusted^1^	
	Correlation coefficient	p value	Correlation coefficient	p value
BMI	0.018	0.58	-0.057	0.092
Waist girth	0.037	0.26	-0.046	0.18
Fasting glucose	0.048	0.14	-0.030	0.36
120 min glucose	0.165	<0.001	0.059	0.084
Total cholesterol	-0.065	0.047	0.012	0.74
LDL cholesterol	-0.060	0.068	-0.047	0.15
HDL cholesterol	-0.094	0.004	-0.080	0.015
Triglycerides	0.057	0.079	-0.007	0.83
SBP	0.068	0.037	-0.001	0.97
DBP	0.046	0.16	-0.005	0.89
Troponin T	0.276	<0.001	0.239	<0.001
NT-proBNP	0.264	<0.001	0.243	<0.001
Cystatin C	0.433	<0.001	0.443	<0.001
CRP	0.178	<0.001	0.152	<0.001

^1^ Adjusted for hypertension, type 2 diabetes, and (where applicable) current smoking, LDL and HDL cholesterol.

### Relations to long-term total mortality

**Figure 1:**
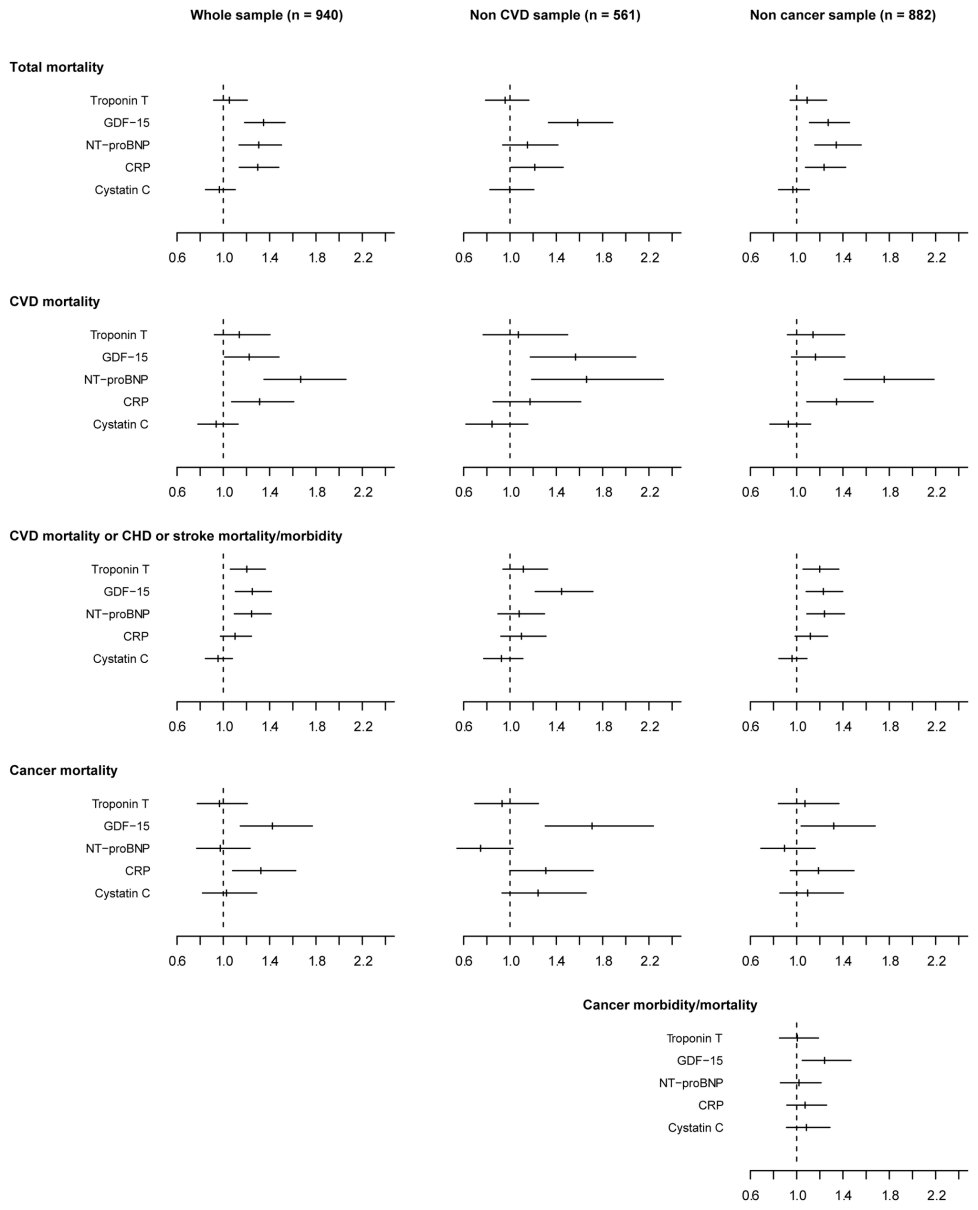
GDF-15 and other biomarkers in relation to cardiovascular and cancer outcomes. Adjusted hazard ratios with 95% CI for 1 SD increase of log transformed GDF-15 values and of log transformed values of other biomarkers in relation to 10 years outcome of total mortality, cardiovascular disease (CVD) mortality, CVD mortality or coronary heart disease (CHD) or stroke morbidity/mortality, cancer mortality and cancer morbidity/mortality, in the whole sample (n = 940), subjects without cardiovascular disease (non CVD sample, n = 561) and subjects without cancer disease (non cancer sample, n = 882) at baseline (outcome cancer morbidity/mortality only in the non-cancer sample). Hazard ratios are adjusted for age, current smoking, body mass index, systolic blood pressure, antihypertensive treatment, total cholesterol, HDL cholesterol, lipid-lowering treatment, type 2 diabetes, and cancer at baseline (except in population P3), and log transformed values of the biomarkers GDF‑15, NT-proBNP, troponin T, cystatin C, and CRP.

**Figure 2:**
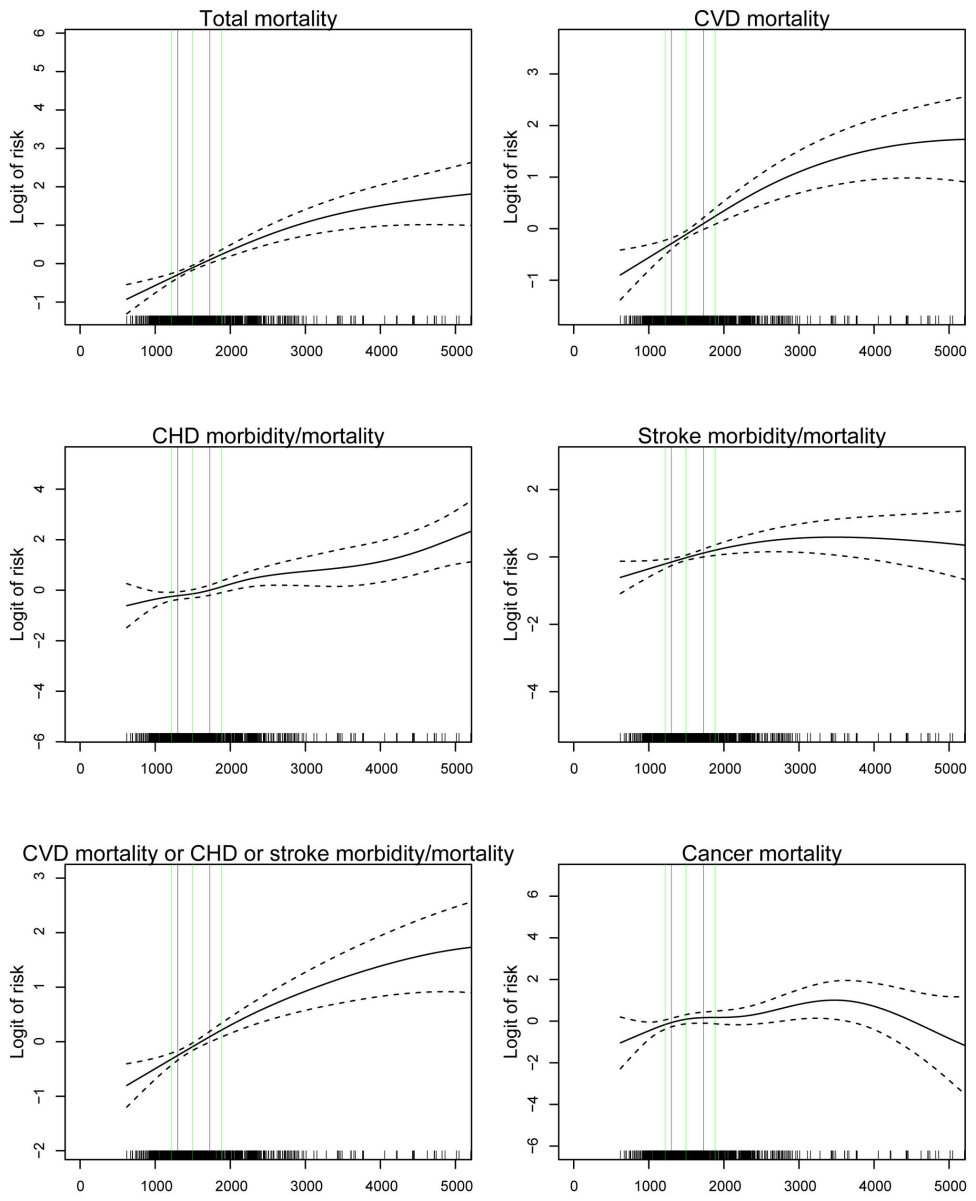
Relations between GDF-15 levels and cardiovascular and cancer outcomes. GDF-15 (ng/l) in relation to logit of risk for total mortality, cardiovascular disease (CVD) mortality, coronary heart disease (CHD) morbidity/mortality, stroke morbidity/mortality, CVD mortality or CHD or stroke morbidity/mortality combined, and cancer mortality.

During follow-up there occurred 265 deaths in the total cohort, 131 in patients without CVD at baseline. Higher GDF-15 levels were significantly and log-linearly related to total mortality (Figure 1). The events were accrued at a fairly stable rate over the ten years follow-up (Figure 2). Per one SD increase in the level of log GDF-15, and after adjustment for conventional cardiovascular risk factors, an increase in total mortality by 48% (95%CI 33 to 67%, p<0.001) and 61% (95%CI 38 to 89%, p<0.001) were observed in the total cohort and the sample without CVD at baseline, respectively, which remained significant also after adjustment for other biomarkers (Table 5). When comparing the impact of the different biomarkers on total mortality, GDF-15 appeared to be a consistent independent prognostic marker of total mortality in the total population as well as in those without CVD or cancer disease at baseline (Figure 1). Also when evaluating the incremental prognostic value for mortality by addition of GDF-15 to conventional risk factors and other biomarkers there was a significant improvement of the integrated discrimination index (IDI) and the net reclassification index (NRI) (Table 6).

**Table 5:** Univariable and multivariable associations between 1 SD increase of the level of log GDF-15 and 10 years outcome concerning total mortality, cardiovascular mortality (CVD), coronary heart disease mortality or morbidity (CHD), stroke mortality or morbidity and the composite of the former events (values are hazard ratios (95 % CI) and p values).

Outcome, events in whole/non-CVD sample	GDF-15 alone		GDF-15 + A1		GDF-15 + A2	
	Whole sample (n = 940)	Non CVD sample (n = 561)	Whole sample (n = 940)	Non CVD sample (n = 561)	Whole sample (n = 940)	Non CVD sample (n = 561)
Total	1.55	1.69	1.48	1.61	1.35	1.58
mortality, 265/131	(1.40, 1.71) p<0.001	(1.46, 1.96) p<0.001	(1.33, 1.66) p<0.001	(1.38, 1.89) p<0.001	(1.18, 1.53) p<0.001	(1.33, 1.89) p<0.001
CVD	1.67	1.85	1.48	1.67	1.22	1.57
mortality, 115/46	(1.45, 1.93) p<0.001	(1.46, 2.34) p<0.001	(1.26, 1.73) p<0.001	(1.28, 2.17) p<0.001	(1.01, 1.48) p=0.037	(1.18, 2.08) p=0.002
CHD	1.53	1.53	1.36	1.44	1.30	1.50
morbidity/mortality, 185/86	(1.35, 1.73) p<0.001	(1.26, 1.87) p<0.001	(1.19, 1.56) p<0.001	(1.17, 1.76) p=0.001	(1.11, 1.52) p=0.001	(1.21, 1.87) p<0.001
Stroke	1.37	1.46	1.28	1.41	1.07	1.30
morbidity/mortality, 133/ 62	(1.18, 1.60) p<0.001	(1.15, 1.84) p=0.002	(1.08, 1.51) p=0.004	(1.10, 1.80) p=0.006	(0.89, 1.29) p=0.473	(0.99, 1.69) p=0.055
Combination	1.50	1.56	1.38	1.47	1.25	1.44
of CVD mortality, CHD or stroke morbidity/ mortality, 304/147	(1.36, 1.66) p<0.001	(1.34, 1.82) p<0.001	(1.24, 1.53) p<0.001	(1.25, 1.73) p<0.001	(1.10, 1.41) p<0.001	(1.22, 1.71) p<0.001

A1 = Adjustment for conventional risk factors, i.e., age, current smoking, body mass index, systolic blood pressure, antihypertensive treatment, total cholesterol, HDL cholesterol, lipid-lowering treatment, type 2 diabetes and (where applicable) previous cancer.

A2 = Adjusted also for levels of other biomarkers – Troponin T, NT-proBNP, CRP and Cystatin C.

**Table 6:** Added predictive capacity of log GDF-15 for 10 years outcome concerning total mortality, cardiovascular mortality (CVD), coronary heart disease mortality or morbidity (CHD), stroke mortality or morbidity and the composite of the former events (values are c statistics, category-free net reclassification improvement (NRI) and integrated discrimination improvement (IDI) for models without and with GDF-15 (c1 and c2 for models without GDF-15 and models with GDF-15, respectively)).

Outcome	Whole sample (n = 940)				Non CVD sample (n = 561)			
	Model A1 + GDF-15		Model A2 + GDF-15		Model A1 + GDF-15		Model A2 + GDF-15	
	C statistics	NRI, IDI	C statistics	NRI, IDI	C statistics	NRI, IDI	C statistics	NRI, IDI
Total mortality	c_1_=0.67 c_2_=0.70 p=0.021	NRI=0.37 p<0.001 IDI=0.039 p<0.001	c_1_=0.71 c_2_=0.73 p=0.027	NRI=0.21 p<0.0036 IDI=0.017 p<0.001	c_1_=0.70 c_2_=0.73 p=0.089	NRI=0.41 p<0.001 IDI=0.051 p<0.001	c_1_=0.71 c_2_=0.74 p=0.066	NRI=0.38 p<0.001 IDI=0.036 p<0.001
CVD morbidity/mortality	c_1_=0.67 c_2_=0.70 p=0.091	NRI=0.36 p<0.001 IDI=0.021 p=0.0016	c_1_=0.75 c_2_=0.76 p=0.12	NRI=0.16 p=0.11 IDI=0.005 p=0.13	c_1_=0.70 c_2_=0.71 p=0.48	NRI=0.40 p=0.0086 IDI=0.033 p=0.0031	c_1_=0.75 c_2_=0.76 p=0.46	NRI=0.35 p=0.023 IDI=0.022 p=0.030
CHD morbidity/mortality	c_1_=0.68 c_2_=0.69 p=0.30	NRI=0.17 p=0.044 IDI=0.018 p<0.001	c_1_=0.70 c_2_=0.71 p=0.21	NRI=0.22 p=0.0069 IDI=0.012 p=0.0037	c_1_=0.70 c_2_=0.71 p=0.32	NRI=0.11 p=0.35 IDI=0.016 p=0.016	c_1_=0.72 c_2_=0.73 p=0.32	NRI=0.30 p=0.010 IDI=0.021 p=0.0032
Stroke morbidity/mortality	c_1_=0.63 c_2_=0.64 p=0.26	NRI=0.23 p=0.013 IDI=0.0031 p=0.12	c_1_=0.70 c_2_=0.70 p=0.83	NRI=0.09 p=0.33 IDI<0.001 p=0.82	c_1_=0.62 c_2_=0.64 p=0.27	NRI=0.32 p=0.019 IDI=0.0063 p=0.11	c_1_=0.66 c_2_=0.66 p=0.91	NRI=0.26 p=0.058 IDI=0.031 p=0.26
Combination of CVD mortality, CHD or stroke morbidity/ mortality	c_1_=0.65 c_2_=0.67 p=0.058	NRI=0.28 p<0.001 IDI=0.024 p<0.001	c_1_=0.69 c_2_=0.70 p=0.23	NRI=0.22 p=0.002 IDI=0.0093 p=0.0041	c_1_=0.65 c_2_=0.67 p=0.092	NRI=0.31 p=0.0014 IDI=0.024 p<0.001	c_1_=0.66 c_2_=0.68 p=0.096	NRI=0.26 p=0.0058 IDI=0.019 p=0.0027

A1 = Conventional risk factors, i.e., age, current smoking, body mass index, systolic blood pressure, antihypertensive treatment, total cholesterol, HDL cholesterol, lipid-lowering treatment, type 2 diabetes, and (where applicable) previous cancer.

A2 = A1 and other biomarkers: Troponin T, NT-proBNP, CRP and Cystatin C.

### Relations to long-term cardiovascular morbidity and mortality

**Figure 3:**
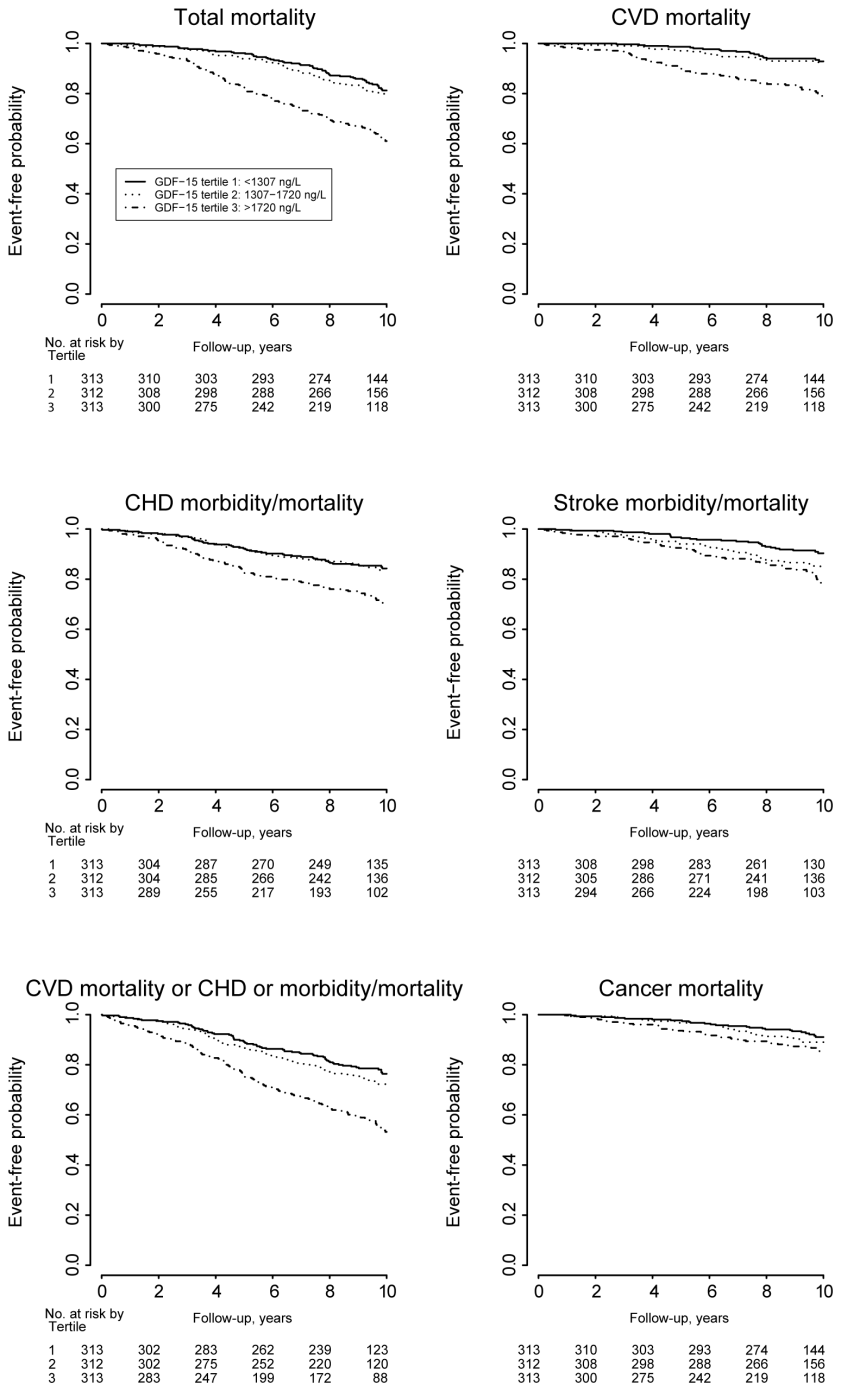
Kaplan-Meier estimates of risk of cardiovascular and cancer outcomes in relation to GDF-15. Kaplan-Meier estimates of event-free probability functions by tertiles of GDF-15 in the total material of 940 subjects for total mortality; cardiovascular disease (CVD) mortality; coronary heart disease (CHD) morbidity or mortality; stroke morbidity or mortality; CVD mortality or CHD or stroke morbidity/mortality; and cancer mortality.

 There were 304 cases with cardiovascular or stroke morbidity or mortality events, including 115 cardiovascular deaths during follow-up out of which 147 respectively 41 cases occurred in the non-cardiovascular disease subgroup. In the total population as well as in the non-CVD population an increasing GDF-15 level was significantly and log-linearly related to total and CVD mortality as well as to CHD and stroke morbidity and mortality and to cancer mortality (Figure 2). The events were accrued at a fairly stable rate over the ten years follow-up (Figure 3). After adjustment for conventional cardiovascular risk factors, one SD increase in the level of log GDF-15 were, in the respective total and non-CVD populations, associated with 48% (95%CI 26 to 73%, p<0.001) and 67% (95%CI 28 to 217%, p<0.001) incremental risk of cardiovascular mortality and 38% (95%CI 24 to 53%, p<0.001) and 47% (95%CI 25 to 73%, p<0.001) increases in CVD mortality and CHD or stroke morbidity and mortality after adjustment for conventional cardiovascular risk factors (Table 5). After adjustment also for other biomarkers the incremental rise in outcome events by one SD increase of log GDF-15 remained significant for all events except stroke. When comparing the impact of the different biomarkers on outcomes in the total population and in cohorts without CVD at entry, GDF-15 was a consistent independent prognostic marker concerning all events (Figure 1). Also when evaluating the incremental prognostic value for cardiovascular morbidity and mortality by addition of GDF-15 to conventional risk factors and other biomarkers, there was a significant improvement of the integrated discrimination index (IDI) and the net reclassification index (NRI) (Table 6).

### Relations to long-term cancer morbidity and mortality

 During follow-up, out of the 940 subjects in the total cohort, 105 deaths were caused by cancer. In the group without cancer at entry (n=882), 182 cases on new cancer morbidity were found, including 85 deaths caused by cancer. During the follow up in the total population as well as in the non-cancer-population an increasing GDF-15 level was significantly related to subsequent cancer mortality (Figure 2). The cancer events accrued at a fairly stable rate over the ten years follow-up (Figure 3). By one SD increase of the level of log GDF-15 there was in the total population a significant 36% (95%CI 15 to 62%, p<0.001) increase in cancer mortality and in the population without cancer disease at baseline an increase of 37% (95%CI 13 to 66%, p<0.001) in cancer mortality and 26% (95%CI 10 to 45%, p<0.001) in cancer morbidity and mortality (Table 7). After adjustments for conventional risk factors, the incremental rise in outcome events by one SD of log GDF-15 remained significant 46% (95%CI 21 to 77%, p<0.001) and 38% (95%CI 12 to 70%, p<0.001) for cancer mortality and 30% (95%CI 12 to 51%, p<0.001) for cancer morbidity and mortality in the respective groups. Only GDF-15 independently predicted cancer morbidity and mortality in the context of all biomarkers (Figure 1). When comparing the impact of the different biomarkers on outcomes, CRP and GDF-15 were the only biomarkers related to cancer morbidity and mortality. When also including the other available baseline characteristics and biomarkers only GDF-15 was significantly related to cancer morbidity and mortality (Figure 1). Sensitivity analyses using lag times of one or two years respectively before counting cancer events gave similar results with maintained statistical significances of results observed. Finally when evaluating the incremental value by addition of GDF-15 to conventional risk factors there was a significant improvement of integrated discrimination index (IDI) and the net reclassification index (NRI) although the significances were somewhat weakened when added also to information from all other biomarkers (Table 8).

**Table 7:** Univariable and multivariable associations between 1 SD increase of the level of log GDF-15 and 10 years outcome concerning cancer mortality and cancer mortality or morbidity (values are hazard ratios (95 % CI) and p values).

Outcome, events in whole/non-cancer sample	GDF-15 alone		GDF-15 + A1		GDF-15 + A2	
	Whole sample (n = 940)	Non cancer sample (n = 882)	Whole sample (n = 940)	Non cancer sample (n = 882)	Whole sample (n = 940)	Non cancer sample (n = 882)
Cancer mortality,	1.36	1.37	1.46	1.38	1.42	1.32
105/85	(1.15, 1.62) p<0.001	(1.13, 1.66) p=0.0012	(1.21, 1.77) p<0.001	(1.12, 1.70) p=0.0025	(1.15, 1.77) p=0.0014	(1.04, 1.68) p=0.023
Cancer morbidity/mortality,		1.26		1.30		1.24
/182		(1.10, 1.45) p<0.001		(1.12, 1.51) p<0.001		(1.05, 1.47) p=0.012

A1 = Adjustment for conventional risk factors, i.e., age, current smoking, body mass index, systolic blood pressure, antihypertensive treatment, total cholesterol, HDL cholesterol, lipid-lowering treatment, type 2 diabetes and (where applicable) previous cancer.

A2 = Adjusted also for levels of other biomarkers – Troponin T, NT-proBNP, CRP and Cystatin C.

**Table 8:** Added predictive capacity of log GDF-15 for 10 years outcome concerning cancer mortality and cancer mortality or morbidity (values are c statistics, category-free net reclassification improvement (NRI) and integrated discrimination improvement (IDI) for models without and with GDF-15 (c1 and c2 for models without GDF-15 and models with GDF-15, respectively)).

Outcome	Whole sample (n = 940)				Non cancer sample (n = 882)			
	Model A1 + GDF-15		Model A2 + GDF-15		Model A1 + GDF-15		Model A2 + GDF-15	
	C statistics	NRI, IDI	C statistics	NRI, IDI	C statistics	NRI, IDI	C statistics	NRI, IDI
Cancer mortality	c_1_=0.65 c_2_=0.67 p=0.29	NRI=0.29 p=0.005 IDI=0.086 p=0.040	c_1_=0.67 c_2_=0.67 p=0.55	NRI=0.17 p=0.093 IDI=0.0062 p=0.095	c_1_=0.61 c_2_=0.63 p=0.28	NRI=0.27 p=0.017 IDI=0.0063 p=0.022	c_1_=0.63 c_2_=0.64 p=0.64	NRI=0.14 p=0.21 IDI=0.0037 p=0.082
Cancer morbidity/mortality					c_1_=0.56 c_2_=0.58 p=0.33	NRI=0.18 p=0.030 IDI=0.0071 p=0.020	c_1_=0.58 c_2_=0.59 p=0.67	NRI=0.12 p=0.17 IDI=0.0034 p=0.10

A1 = Conventional risk factors, i.e., age, current smoking, body mass index, systolic blood pressure, antihypertensive treatment, total cholesterol, HDL cholesterol, lipid-lowering treatment, type 2 diabetes, and (where applicable) previous cancer. A2 = A1 and other biomarkers: Troponin T, NT-proBNP, CRP and Cystatin C.

## Discussion

 In the present study we showed that the level of GDF-15, independent of established clinical risk factors, was prognostic for both cardiovascular and cancer morbidity and mortality over the forthcoming 10 years with a 30-50 % increase in risk for one SD increase of the log GDF-15 level in apparently healthy elderly men. This prognostic information was independent of levels of biomarkers of cardiac and renal dysfunction and inflammation. The association between GDF-15 was the only independent biomarker for cardiovascular and cancer morbidity and mortality in subjects without the respective diseases at baseline. The consistent associations between the GDF-15 level and smoking, diabetes, and biomarkers of myocardial and renal dysfunction and inflammation [13,14,27,28] suggested that expression of GDF-15 might be a shared and early indicator of cellular vulnerability to the development of vascular and cancer diseases. 

From a cohort of 1004 70-year old community-dwelling subjects we previously reported that the GDF-15 level was associated with cardiovascular risk factors, myocardial and renal dysfunction and indicators of inflammation, vascular dysfunction and plaque burden [13,14]. From another cohort of 950 patients stabilized 6 months after an episode of acute coronary syndrome we reported similar association between the GDF-15 level and age, sex, smoking, diabetes mellitus, hypertension, renal function, NT-proBNP and troponin T levels and also the associations to raised cardiovascular morbidity and mortality during 5 years follow-up [17]. Combining one cohort including 976 men, 47 to 80 years of age, with 5.3 years of follow-up with another cohort of 324 subjects, mainly female twins age 63 to 93, with 9 years of follow-up, Wiklund et al. reported that high GDF-15 levels were associated with as well cardiovascular as cancer mortality [26]. In a community cohort of 1391 subjects, with a mean age of 70 years at entry, the level of GDF-15 had similar associations to cardiovascular risk factors, renal and myocardial dysfunction and was independently related to both cardiovascular and non-cardiovascular mortality during 11 years of follow-up [27]. Also in a cohort of 3219 adults 30-65 years of age a high GDF-15-level was associated with age, hypertension, cardiovascular risk factors, cardiac and renal dysfunction, coronary calcification and was independently related to all cause and cardiovascular mortality during 7 years follow-up [28]. Finally in a Framingham offspring cohort of 3428 men or women with average age of 59 years followed for an average of 11 years, GDF-15 appeared the strongest prognostic marker for total mortality although several other biomarkers (troponin-I, soluble ST2 and BNP) were equally prognostic concerning all cardiovascular events [30]. 

Several epidemiological studies have highlighted that cardiovascular and cancer diseases have many risk factors in common e.g. age, smoking, obesity, diabetes, and dietary habits [1,2]. There are also treatments that seem to prevent both cancer and CVD e.g. aspirin [34]. Concerning the diagnosis and prognosis of specific cancer diseases a variety of biomarkers are available [35,36]. There are however few biomarkers indicating a generally raised risk of cancer morbidity and mortality in currently healthy subjects. Previously the level of cystatin C has been associated with cardiovascular and as well as cancer mortality in healthy subjects [37,38], which however, could not be sustained in the present multivariable analyses. Cathepsin-S has recently been found associated with both cardiovascular and cancer mortality in the present cohort, although not including adjustment for biomarkers including troponin T and NT-proBNP and GDF-15 [39]. The association between GDF-15 and future cardiovascular and cancer morbidity and mortality seems very robust, especially as it is most prominent in participants without previous cardiovascular or cancer disease. The lack of association between GDF-15 at baseline and a cancer diagnosis at baseline the ULSAM cohort are probably related to that these diagnoses relate to previously successfully treated cancer disease. In the performed sensitivity analyses, excluding also patients with a cancer diagnosis during the first year and two years respectively, no apparent changes in the point estimates for future cancer events were observed. Thus, it is unlikely that cancer present already at baseline was a driver of the results obtained.

Several mechanisms might explain the association between the GDF-15 level and cardiovascular and cancer morbidity and mortality. GDF-15 is expressed by many cell types in response to oxidative stress and inflammation and seems to be involved in the regulation of apoptosis, cell proliferation and cellular repair, biological processes that are key components of cardiovascular and cancer pathobiology [7,40]. Human GDF-15 expression is controlled by p53 which is linked to atherosclerosis and cancer [41]. GDF-15 is expressed by myocardial cells at ischemia, injury, reperfusion [11,42] and cardiac dysfunction [8,9], and also by monocytes/macrophages involved in the atherosclerosis process [43,44]. Both inflammatory processes and several cancer diseases can lead to the expression of GDF-15 in several cell types [45]. These processes are more common at higher age, male sex, smoking, obesity, and diabetes mellitus, contributing to the association between GDF-15 level and cardiovascular and cancer diseases. Elevation of the GDF-15 level might appear as an expression of cellular stress, dysfunction, aging and the need for repair even before any specific organ disease has developed. Therefore, elevation of GDF-15 level might be a reason for further investigations and/or strengthen the indication for preventive measures against both CVD and cancer. 

There were some limitations with the study. Genetic predisposition to higher GDF-15 levels might be associated with reduced long-levity was not investigated in the current study but found no support the previously reported twin study [26]. The study only contained elderly white males in one Scandinavian country and might therefore not be relevant to other populations. There was a lack of information on some recognized risk factors for cancer e.g. heredity, precancerous conditions etc. Although the follow-up of all patients was complete through public registries there was no central adjudication or other validation of cardiovascular or cancer outcome events. 

## References

[1] Perk J, De Backer G, Gohlke H, Graham I, Reiner Z et al. (2012) European Guidelines on cardiovascular disease prevention in clinical practice (version 2012): The Fifth Joint Task Force of the European Society of Cardiology and Other Societies on Cardiovascular Disease Prevention in Clinical Practice (constituted by representatives of nine societies and by invited experts) * Developed with the special contribution of the European Association for Cardiovascular Prevention & Rehabilitation (EACPR). Eur Heart J 33: 1635-1701. doi:10.1093/eurheartj/ehs092. PubMed: 22555213. 22555213

[2] Romaguera D, Vergnaud AC, Peeters PH, van Gils CH, Chan DS et al. (2012) Is concordance with World Cancer Research Fund/American Institute for Cancer Research guidelines for cancer prevention related to subsequent risk of cancer? Results from the EPIC study. Am J Clin Nutr 96: 150-163. doi:10.3945/ajcn.111.031674. PubMed: 22592101. 22592101

[3] Zethelius B, Berglund L, Sundström J, Ingelsson E, Basu S et al. (2008) Use of multiple biomarkers to improve the prediction of death from cardiovascular causes. N Engl J Med 358: 2107-2116. doi:10.1056/NEJMoa0707064. PubMed: 18480203. 18480203

[4] Blankenberg S, Zeller T, Saarela O, Havulinna AS, Kee F et al. (2010) Contribution of 30 biomarkers to 10-year cardiovascular risk estimation in 2 population cohorts: the MONICA, risk, genetics, archiving, and monograph (MORGAM) biomarker project. Circulation 121: 2388-2397. doi:10.1161/CIRCULATIONAHA.109.901413. PubMed: 20497981. 20497981

[5] Saunders JT, Nambi V, de Lemos JA, Chambless LE, Virani SS et al. (2011) Cardiac troponin T measured by a highly sensitive assay predicts coronary heart disease, heart failure, and mortality in the Atherosclerosis Risk in Communities Study. Circulation 123: 1367-1376. doi:10.1161/CIRCULATIONAHA.110.005264. PubMed: 21422391. 21422391 PMC3072024

[6] Xu R, Ye P, Luo L, Xiao W, Sheng L et al. (2011) Association between high-sensitivity cardiac troponin T and predicted cardiovascular risks in a community-based population. Int J Cardiol 149: 253-256. doi:10.1016/j.ijcard.2011.02.032. PubMed: 21388695. 21388695

[7] Breit SN, Johnen H, Cook AD, Tsai VW, Mohammad MG et al. (2011) The TGF-beta superfamily cytokine, MIC-1/GDF15: a pleotrophic cytokine with roles in inflammation, cancer and metabolism. Growth Factors 29: 187-195. doi:10.3109/08977194.2011.607137. PubMed: 21831009. 21831009

[8] Wollert KC, Kempf T (2012) Growth Differentiation Factor 15 in Heart Failure: An. Update - Curr Heart Fail Rep 9: 337-345. doi:10.1007/s11897-012-0113-9. 22961192

[9] Kempf T, von Haehling S, Peter T, Allhoff T, Cicoira M et al. (2007) Prognostic utility of growth differentiation factor-15 in patients with chronic heart failure. J Am Coll Cardiol 50: 1054-1060. doi:10.1016/j.jacc.2007.04.091. PubMed: 17825714. 17825714

[10] Kempf T, Horn-Wichmann R, Brabant G, Peter T, Allhoff T et al. (2007) Circulating concentrations of growth-differentiation factor 15 in apparently healthy elderly individuals and patients with chronic heart failure as assessed by a new immunoradiometric sandwich assay. Clin Chem 53: 284-291. PubMed: 17185363. 17185363 10.1373/clinchem.2006.076828

[11] Kempf T, Eden M, Strelau J, Naguib M, Willenbockel C et al. (2006) The transforming growth factor-beta superfamily member growth-differentiation factor-15 protects the heart from ischemia/reperfusion injury. Circ Res 98: 351-360. doi:10.1161/01.RES.0000202805.73038.48. PubMed: 16397141. 16397141

[12] Kempf T, Wollert KC (2009) Growth differentiation factor-15: a new biomarker in cardiovascular disease. Herz 34: 594-599. doi:10.1007/s00059-009-3317-3. PubMed: 20024638. 20024638

[13] Lind L, Wallentin L, Kempf T, Tapken H, Quint A et al. (2009) Growth-differentiation factor-15 is an independent marker of cardiovascular dysfunction and disease in the elderly: results from the Prospective Investigation of the Vasculature in Uppsala Seniors (PIVUS) Study. Eur Heart J 30: 2346-2353. doi:10.1093/eurheartj/ehp261. PubMed: 19561023. 19561023

[14] Eggers KM, Kempf T, Lind L, Sundström J, Wallentin L et al. (2012) Relations of growth-differentiation factor-15 to biomarkers reflecting vascular pathologies in a population-based sample of elderly subjects. Scand J Clin Lab Invest 72: 45-51. doi:10.3109/00365513.2011.626072. PubMed: 22023041. 22023041

[15] Wollert KC, Kempf T, Peter T, Olofsson S, James S et al. (2007) Prognostic value of growth-differentiation factor-15 in patients with non-ST-elevation acute coronary syndrome. Circulation 115: 962-971. doi:10.1161/CIRCULATIONAHA.106.650846. PubMed: 17283261. 17283261

[16] Eggers KM, Kempf T, Allhoff T, Lindahl B, Wallentin L et al. (2008) Growth-differentiation factor-15 for early risk stratification in patients with acute chest pain. Eur Heart J 29: 2327-2335. doi:10.1093/eurheartj/ehn339. PubMed: 18664460. 18664460 PMC2556729

[17] Eggers KM, Kempf T, Lagerqvist B, Lindahl B, Olofsson S et al. (2010) Growth-differentiation factor-15 for long-term risk prediction in patients stabilized after an episode of non-ST-segment-elevation acute coronary syndrome. Circ Cardiovasc Genet 3: 88-96. doi:10.1161/CIRCGENETICS.109.877456. PubMed: 20160200. 20160200

[18] Anand IS, Kempf T, Rector TS, Tapken H, Allhoff T et al. (2010) Serial measurement of growth-differentiation factor-15 in heart failure: relation to disease severity and prognosis in the Valsartan Heart Failure Trial. Circulation 122: 1387-1395. doi:10.1161/CIRCULATIONAHA.109.928846. PubMed: 20855664. 20855664

[19] Brown DA, Hance KW, Rogers CJ, Sansbury LB, Albert PS et al. (2012) Serum macrophage inhibitory cytokine-1 (MIC-1/GDF15): a potential screening tool for the prevention of colon cancer? Cancer Epidemiol Biomarkers Prev 21: 337-346. doi:10.1158/1055-9965.EPI-11-0786. PubMed: 22144502. 22144502 PMC7238292

[20] Wallin U, Glimelius B, Jirström K, Darmanis S, Nong RY et al. (2011) Growth differentiation factor 15: a prognostic marker for recurrence in colorectal cancer. Br J Cancer 104: 1619-1627. doi:10.1038/bjc.2011.112. PubMed: 21468045. 21468045 PMC3101900

[21] Corre J, Labat E, Espagnolle N, Hébraud B, Avet-Loiseau H et al. (2012) Bioactivity and prognostic significance of growth differentiation factor GDF15 secreted by bone marrow mesenchymal stem cells in multiple myeloma. Cancer Res 72: 1395-1406. doi:10.1158/1538-7445.AM2012-1395. PubMed: 22301101. 22301101

[22] Brown DA, Lindmark F, Stattin P, Bälter K, Adami HO et al. (2009) Macrophage inhibitory cytokine 1: a new prognostic marker in prostate cancer. Clin Cancer Res 15: 6658-6664. doi:10.1158/1078-0432.CCR-08-3126. PubMed: 19843661. 19843661 PMC3557964

[23] Staff AC, Bock AJ, Becker C, Kempf T, Wollert KC et al. (2010) Growth differentiation factor-15 as a prognostic biomarker in ovarian cancer. Gynecol Oncol 118: 237-243. doi:10.1016/j.ygyno.2010.05.032. PubMed: 20576287. 20576287

[24] Brown DA, Ward RL, Buckhaults P, Liu T, Romans KE et al. (2003) MIC-1 serum level and genotype: associations with progress and prognosis of colorectal carcinoma. Clin Cancer Res 9: 2642-2650. PubMed: 12855642. 12855642

[25] Welsh JB, Sapinoso LM, Kern SG, Brown DA, Liu T et al. (2003) Large-scale delineation of secreted protein biomarkers overexpressed in cancer tissue and serum. Proc Natl Acad Sci U S A 100: 3410-3415. doi:10.1073/pnas.0530278100. PubMed: 12624183. 12624183 PMC152306

[26] Wiklund FE, Bennet AM, Magnusson PK, Eriksson UK, Lindmark F et al. (2010) Macrophage inhibitory cytokine-1 (MIC-1/GDF15): a new marker of all-cause mortality. Aging Cell 9: 1057-1064. doi:10.1111/j.1474-9726.2010.00629.x. PubMed: 20854422. 20854422 PMC4139960

[27] Daniels LB, Clopton P, Laughlin GA, Maisel AS, Barrett-Connor E (2011) Growth-differentiation factor-15 is a robust, independent predictor of 11-year mortality risk in community-dwelling older adults: the Rancho Bernardo Study. Circulation 123: 2101-2110. doi:10.1161/CIRCULATIONAHA.110.979740. PubMed: 21536998. 21536998 PMC3107013

[28] Rohatgi A, Patel P, Das SR, Ayers CR, Khera A et al. (2012) Association of growth differentiation factor-15 with coronary atherosclerosis and mortality in a young, multiethnic population: observations from the Dallas Heart Study. Clin Chem 58: 172-182. doi:10.1373/clinchem.2011.171926. PubMed: 22065155. 22065155 PMC3926660

[29] Brown DA, Breit SN, Buring J, Fairlie WD, Bauskin AR et al. (2002) Concentration in plasma of macrophage inhibitory cytokine-1 and risk of cardiovascular events in women: a nested case-control study. Lancet 359: 2159-2163. doi:10.1016/S0140-6736(02)09093-1. PubMed: 12090982. 12090982

[30] Wang TJ, Wollert KC, Larson MG, Coglianese E, McCabe EL et al. (2012) Prognostic utility of novel biomarkers of cardiovascular stress: the Framingham heart study. Circulation 126: 1596-1604. doi:10.1161/CIRCULATIONAHA.112.129437. PubMed: 22907935. 22907935 PMC3656719

[31] Pencina MJ, D'Agostino RB (2004) Overall C as a measure of discrimination in survival analysis: model specific population value and confidence interval estimation. Stat Med 23: 2109-2123. doi:10.1002/sim.1802. PubMed: 15211606. 15211606

[32] Pencina MJ, D'Agostino RB Sr, D'Agostino RB Jr, Vasan RS (2008) Evaluating the added predictive ability of a new marker: from area under the ROC curve to reclassification and beyond. Stat Med 27: 157-212; discussion: 17569110. 17569110 10.1002/sim.2929

[33] Pencina MJ, D'Agostino RB Sr, Steyerberg EW (2011) Extensions of net reclassification improvement calculations to measure usefulness of new biomarkers. Stat Med 30: 11-21. doi:10.1002/sim.4085. PubMed: 21204120. 21204120 PMC3341973

[34] Rothwell PM, Price JF, Fowkes FG, Zanchetti A, Roncaglioni MC et al. (2012) Short-term effects of daily aspirin on cancer incidence, mortality, and non-vascular death: analysis of the time course of risks and benefits in 51 randomised controlled trials. Lancet 379: 1602-1612. doi:10.1016/S0140-6736(11)61720-0. PubMed: 22440946. 22440946

[35] Henry NL, Hayes DF (2012) Cancer biomarkers. Mol Oncol. 6: 140-146. doi:10.1016/j.molonc.2012.01.010. PubMed: 22356776. 22356776 PMC5528374

[36] Verma M (2012) Epigenetic biomarkers in cancer epidemiology. Methods Mol Biol 863: 467-480. doi:10.1007/978-1-61779-612-8_28. PubMed: 22359311. 22359311

[37] Shlipak MG, Sarnak MJ, Katz R, Fried LF, Seliger SL et al. (2005) Cystatin C and the risk of death and cardiovascular events among elderly persons. N Engl J Med 352: 2049-2060. doi:10.1056/NEJMoa043161. PubMed: 15901858. 15901858

[38] Wu CK, Lin JW, Caffrey JL, Chang MH, Hwang J.Jet al (2010) Cystatin C and long-term mortality among subjects with normal creatinine-based estimated glomerular filtration rates: NHANES III (Third National Health and Nutrition Examination Survey). J Am Coll Cardiol 56:1930-6

[39] Jobs E, Ingelsson E, Risérus U, Nerpin E, Jobs M et al. (2011) Association between serum cathepsin S and mortality in older adults. JAMA 306: 1113-1121. doi:10.1001/jama.2011.1246. PubMed: 21878432. 21878432

[40] Xu X, Li Z, Gao W (2011) Growth differentiation factor 15 in cardiovascular diseases: from bench to bedside. Biomarkers 16: 466-475. doi:10.3109/1354750X.2011.580006. PubMed: 21718220. 21718220

[41] Yang H, Filipovic Z, Brown D, Breit SN, Vassilev LT (2003) Macrophage inhibitory cytokine-1: a novel biomarker for p53 pathway activation. Mol Cancer Ther 2: 1023-1029. PubMed: 14578467. 14578467

[42] Kempf T, Zarbock A, Widera C, Butz S, Stadtmann A et al. (2011) GDF-15 is an inhibitor of leukocyte integrin activation required for survival after myocardial infarction in mice. Nat Med 17: 581-588. doi:10.1038/nm.2354. PubMed: 21516086. 21516086

[43] de Jager SC, Bermúdez B, Bot I, Koenen RR, Bot M et al. (2011) Growth differentiation factor 15 deficiency protects against atherosclerosis by attenuating CCR2-mediated macrophage chemotaxis. J Exp Med 208: 217-225. doi:10.1084/jem.20100370. PubMed: 21242297. 21242297 PMC3039852

[44] Schlittenhardt D, Schober A, Strelau J, Bonaterra GA, Schmiedt W et al. (2004) Involvement of growth differentiation factor-15/macrophage inhibitory cytokine-1 (GDF-15/MIC-1) in oxLDL-induced apoptosis of human macrophages in vitro and in arteriosclerotic lesions. Cell Tissue Res 318: 325-333. doi:10.1007/s00441-004-0986-3. PubMed: 15459768. 15459768

[45] Mimeault M, Batra SK (2010) Divergent molecular mechanisms underlying the pleiotropic functions of macrophage inhibitory cytokine-1 in cancer. J Cell Physiol 224: 626-635. doi:10.1002/jcp.22196. PubMed: 20578239. 20578239 PMC2932466

